# Supplementary material for: Activation of endogenous retroviruses during brain development causes an inflammatory response
Source: EMBO J. 2021 Mar 1;40(9):e106423. doi: 10.15252/embj.2020106423 (PMC8090857; doi:10.15252/embj.2020106423)
Supplement: Supplementary file 1 — Expanded View Figures PDF [file EMBJ-40-e106423-s003.pdf]

Expanded View Figures

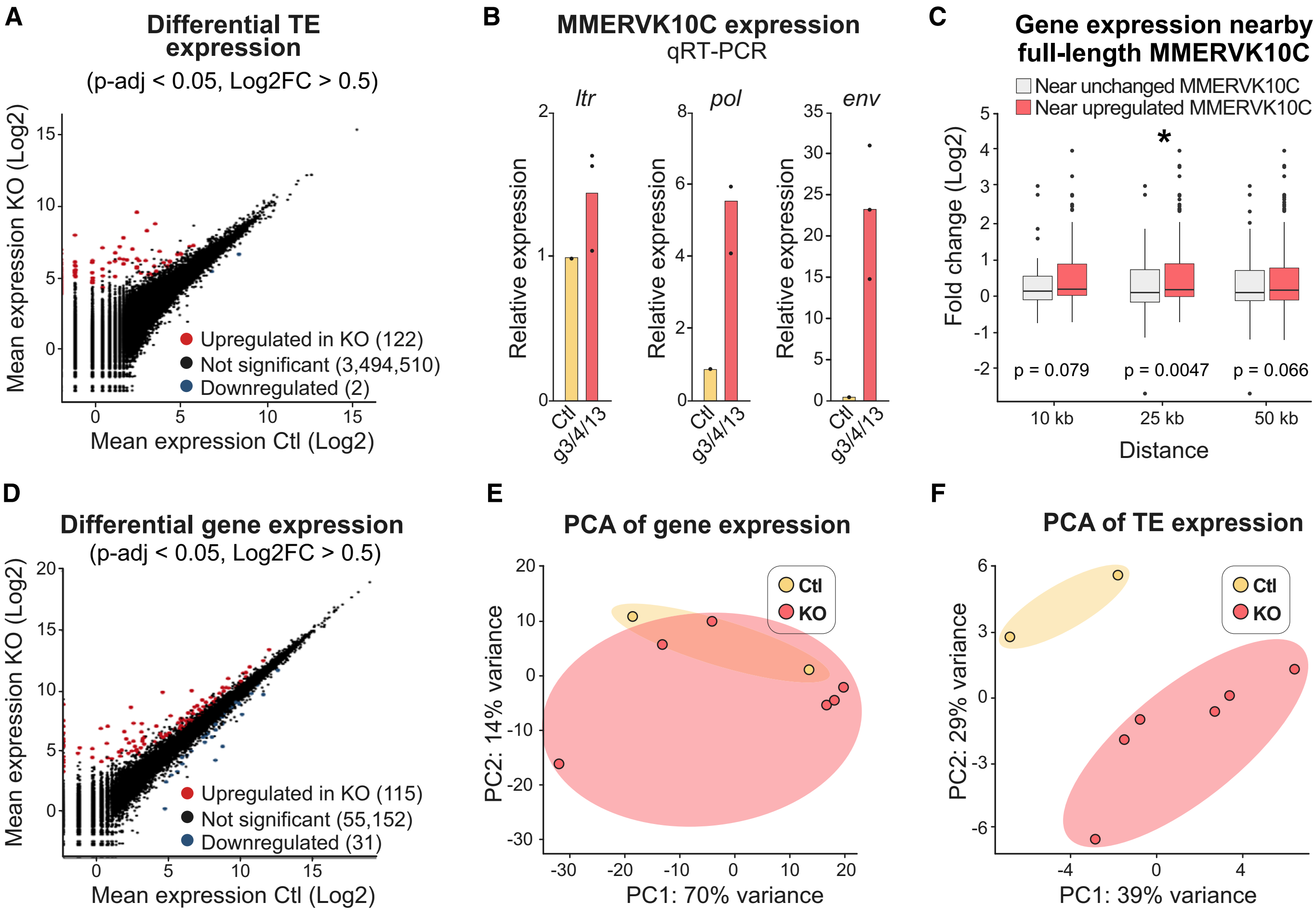

Figure EV1. CRISPR/Cas9 deletion of *Trim28* in NPCs results in upregulation of ERVs and nearby genes.

A Differential expression analysis of individual TEs in mouse NPCs upon CRISPR/Cas9-mediatied disruption of *Trim28* was performed with DESeq2 (described in Materials and Methods).

B The upregulated transcription of MMERVK10C elements was validated by qRT–PCR. Columns show an average of the individual data points.

C Genes located in the close vicinity of upregulated MMERVK10C elements were significantly upregulated. X-axis indicate the window-of-inclusion for genes located close to an MMERVK10C. Boxplot hinges represent first and third quartile, and the median is indicated by the central band. Whiskers extend to 1.5 times the interquartile range.

D RNA-seq differential expression analysis of protein coding genes performed with DESeq2 (described in Materials and Methods) in mouse NPCs upon CRISPR/Cas9-mediatied disruption of *Trim28*.

E, F PCA analysis of gene expression was unable to distinguish the *Trim28*-KO cells from Ctl, while PCA analysis of TE expression was. The spread of the groups is visualized with opaque ellipses.

Source data are available online for this figure.

© 2021 The Authors

Figure EV2. Deletion of *Trim28* in adult neurons *in vivo* does not result in an upregulation of ERVs.

A A schematic of the workflow targeting *Trim28* in the forebrain of Stop-Cas9-GFP knock-in mice using AAV vectors expressing the gRNA and a nuclear RFP reporter as well as an AAV vector expressing Cre, specifically in neurons by the Synapsin promoter. 8 weeks after injection, the animals were analyzed either by IHC or by DNA/RNA-sequencing following nuclei isolation by FACS.

B Gene editing efficiency was evaluated by amplicon sequencing of the respective targeted sequences. DNA was isolated from 50,000 RFP^{+} nuclei per animal, one animal per group was analyzed. Black bars indicate % of frameshift mutations.

C, D Neuron-specific editing of the *Trim28*-loci resulted in a robust loss of the Trim28 protein in neurons, as evaluated by IHC where the expression of Trim28 in RFP^{+} cells was quantified and is displayed as mean ± SEM. Approximately 550 RFP^{+}/GFP^{+} cells per animal and group was evaluated. Scale bar 30 μm.

E Differential expression analysis of TEs in adult neurons from the Stop-Cas9-GFP knock-in mice upon *Trim28*-KO was done by using TEtranscripts and DESeq2 (described in Materials and Methods).

F A schematic of the workflow targeting *Trim28* in adult neurons in the forebrain of *Trim28*-flox mice (+/− and +/+) by co-injecting AAV vectors expressing Cre or nuclear GFP under the control of the Synapsin promoter. 8 weeks after injection, the animals were analyzed either by IHC or by RNA-sequencing following nuclei isolation by FACS.

G RNA-seq analysis of the isolated GFP^{+} nuclei revealed a loss of the *Trim28* transcript. Boxplot hinges represent first and third quartile, and the median is indicated by the central band. Whiskers extend to 1.5 times the interquartile range.

H TE differential expression analysis on the adult neurons in the *Trim28*-flox animals was done using TEtranscripts and DESeq2 (described in Materials and Methods).

I IHC for Trim28, GFP, and the neuronal marker NeuN revealed a complete neuronal loss of the protein Trim28 in the *Trim28*-KO animals (*Trim28*-flox mice (+/+)). Scale bar 75 μm.

 © 2021 The Authors

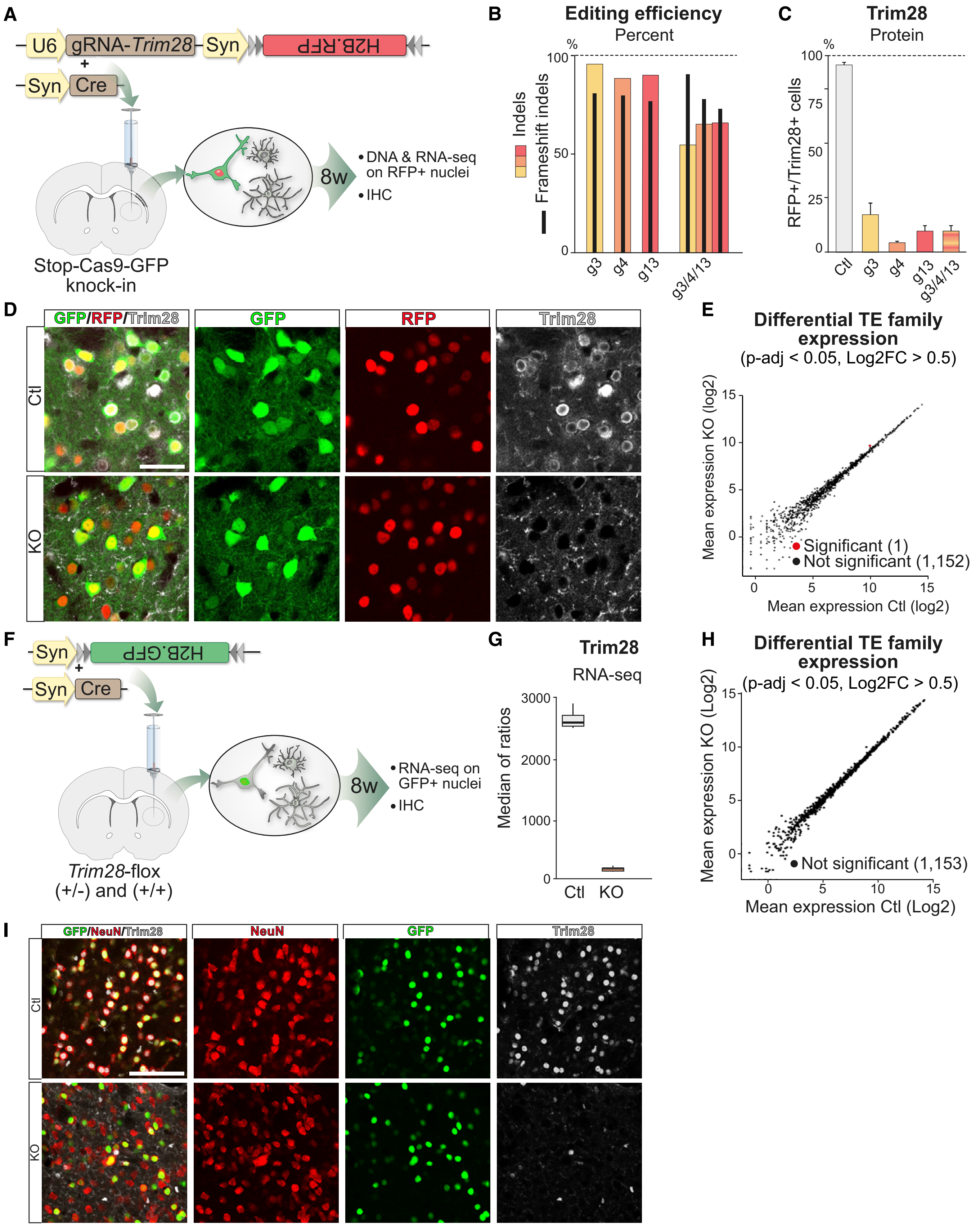
A
U6
gRNA-Trim28
Syn
H2B.RFP
+
Syn
Cre
DNA & RNA-seq on RFP+ nuclei
IHC
8w
Stop-Cas9-GFP knock-in
B
Editing efficiency
Percent
%
Indels
Frameshift indels
g3
g4
g13
g3/4/13
C
Trim28
Protein
RFP+/Trim28+ cells
Ctl
D
GFP/RFP/Trim28
GFP
RFP
Trim28
Ctl
KO
E
Differential TE family expression
(p-adj < 0.05, Log2FC > 0.5)
Mean expression KO (log2)
Mean expression Ctl (log2)
Significant (1)
Not significant (1,152)
F
Syn
H2B.GFP
Cre
RNA-seq on GFP+ nuclei
Trim28-flox (+/-) and (+/+)
G
Trim28
RNA-seq
Median of ratios
KO
H
Mean expression KO (Log2)
Mean expression Ctl (Log2)
Not significant (1,153)
I
GFP/NeuN/Trim28
NeuN

Figure EV2.

Figure EV3. Differential gene expression upon loss of *Trim28*.

A RNA-seq analysis of *Trim28* expression in the adult cortex of *Emx1*-Cre/ *Trim28*-KO animals (*Emx1*-Cre(+/−), *Trim28*-flox(+/+)). Boxplot hinges represent first and third quartile, and the median is indicated by the central band. Whiskers extend to 1.5 times the interquartile range.

B Differential TE expression analysis for individual TEs in the adult cortex of *Emx1*-Cre/*Trim28*-KO animals compared to their Cre-negative control litter mates was performed with DESeq2 (described in Materials and Methods).

C Expression of genes nearby upregulated full length MMERVK10Cs were not upregulated in the *Emx1*-Cre/*Trim28*-KO animals. X-axis indicate the window-of-inclusion for genes located close to an MMERVK10C (test performed with DESeq2, Log2FC > 0). Boxplot hinges represent first and third quartile, and the median is indicated by the central band. Whiskers extend to 1.5 times the interquartile range.

D No transcriptional readthrough into nearby genes from upregulated MMERVK10Cs were observed, exemplified with an UCSC screenshot of the same location in the genome as shown in Fig 1G.

E Differential gene expression performed with DESeq2 (see Materials and Methods) in the adult cortex of *Emx1*-Cre/*Trim28*-KO animals compared to their control litter mates.

F Differential gene expression performed with DESeq2 (see Materials and Methods) in the adult forebrain upon AAV.Cre/*Trim28*-KO compared to controls.

G Venn diagrams showing significantly up- and downregulated genes in the *Emx1*-Cre/*Trim28*-KO and the AAV.Cre/*Trim28*-KO animals, as well as the genes overlapping in between them.

H GO term analysis of the overlapping upregulated genes between *Emx1*-Cre/*Trim28*-KO and the AAV.Cre/*Trim28*-KO animals.

© 2021 The Authors

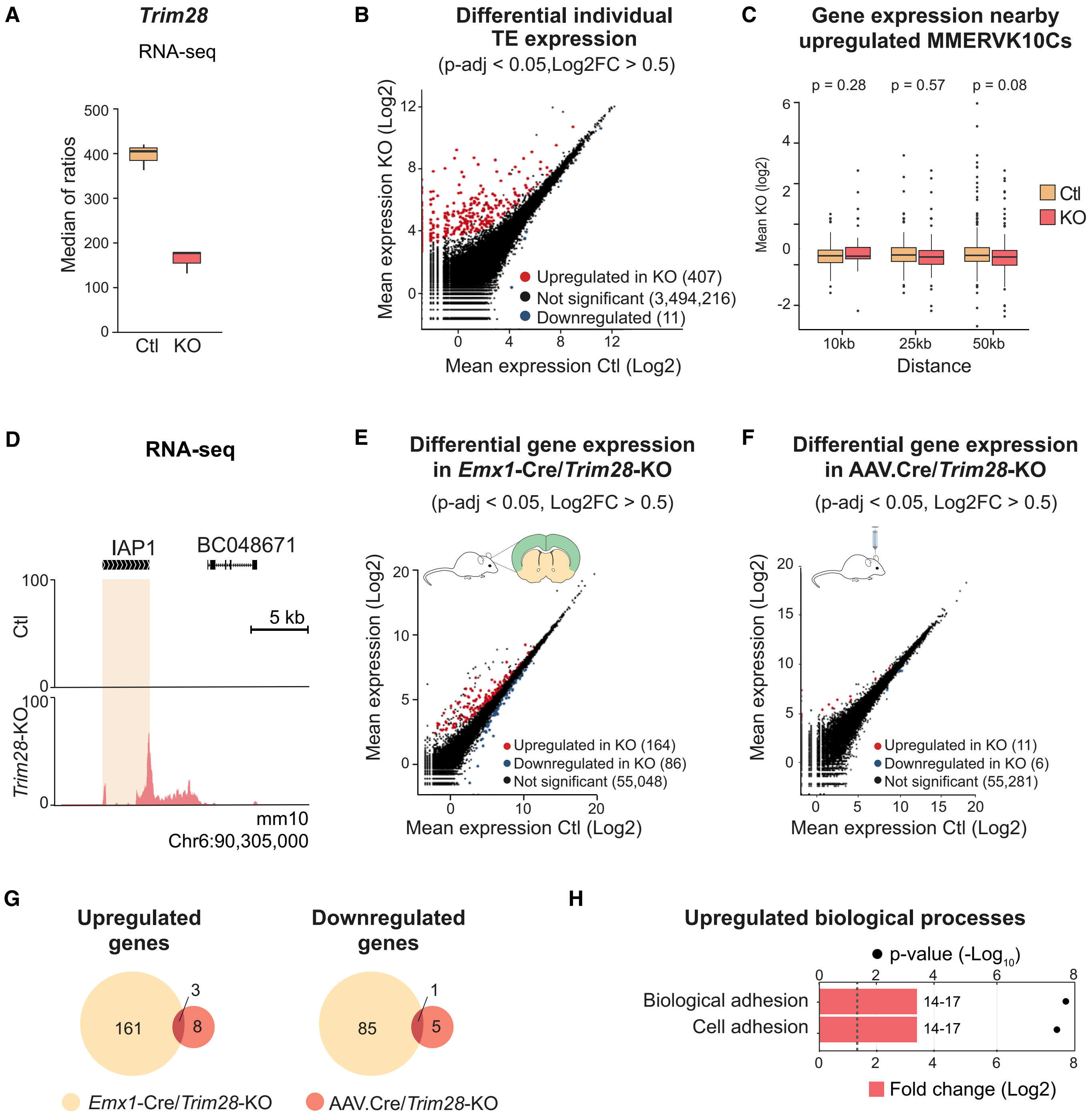
A
Trim28
RNA-seq
Median of ratios
Ctl KO
B
Differential individual TE expression
(p-adj < 0.05,Log2FC > 0.5)
Mean expression KO (Log2)
Mean expression Ctl (Log2)
Upregulated in KO (407)
Not significant (3,494,216)
Downregulated (11)
C
Gene expression nearby upregulated MMERVK10Cs
p = 0.28 p = 0.57 p = 0.08
Mean KO (log2)
10kb 25kb 50kb
Distance
Ctl
KO
D
RNA-seq
IAP1
BC048671
5 kb
Ctl
Trim28-KO
mm10
Chr6:90,305,000
E
Differential gene expression in Emx1-Cre/Trim28-KO
(p-adj < 0.05, Log2FC > 0.5)
Mean expression (Log2)
Mean expression Ctl (Log2)
Upregulated in KO (164)
Downregulated in KO (86)
Not significant (55,048)
F
Differential gene expression in AAV.Cre/Trim28-KO
(p-adj < 0.05, Log2FC > 0.5)
Mean expression (Log2)
Mean expression Ctl (Log2)
Upregulated in KO (11)
Downregulated in KO (6)
Not significant (55,281)
G
Upregulated genes
161 3 8
Downregulated genes
85 1 5
Emx1-Cre/Trim28-KO
AAV.Cre/Trim28-KO
H
Upregulated biological processes
p-value (-Log10)
Biological adhesion 14-17
Cell adhesion 14-17
Fold change (Log2)

Figure EV3.

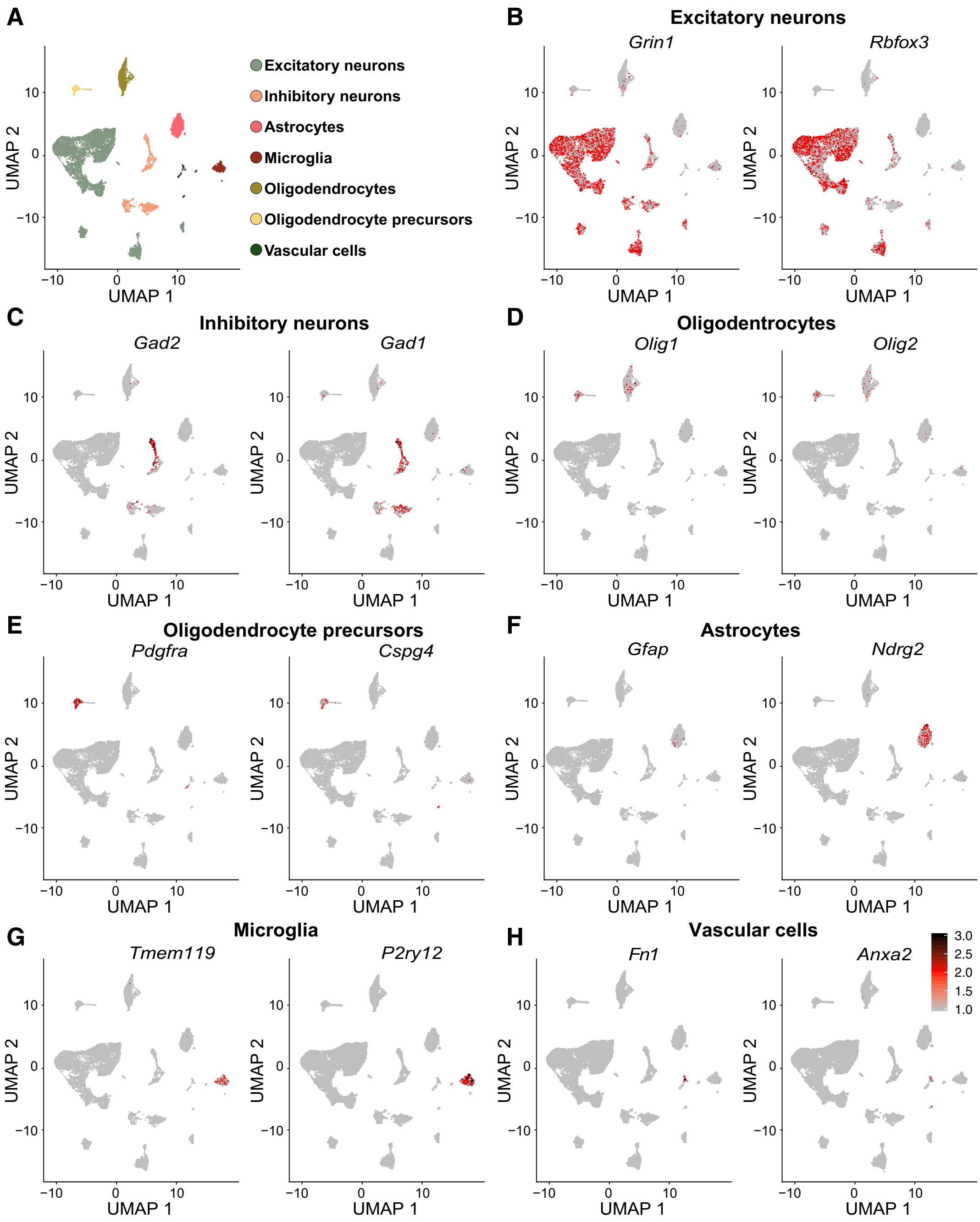

Figure EV4. Cell type marker expression projected onto the UMAP plot of *Emx1*-Cre/*Trim28* cell clusters.

A An overview of the defined cell clusters from single cell RNA-seq of *Emx1*-Cre/*Trim28*-flox animals in an UMAP plot.

B–H The projected expression of two cell markers per cluster on the UMAP.

Source data are available online for this figure.

 © 2021 The Authors

Figure EV5. Iba1$^+$ densities and inflammatory/viral defense genes upon *Trim28*-KO.

A The number of Iba1$^+$ cells did not differ in the cortex of *Emx1*-Cre/*Trim28*-KO animals and controls, here shown as mean ± SEM, unpaired *t*-test. Iba1$^+$ cells were counted in 20 photographs (20x objective) per animal, control $n = 3$ and KO $n = 2$.

B Immunohistochemical analysis for the microglia marker upon *Trim28*-KO in mature neurons *in vivo* (AAV.Syn-Cre/*Trim28*-KO animals) revealed no difference in Iba1 morphology between *Trim28*-KO animals and controls. Scale bar 75 μm.

C Inflammatory genes were chosen from 216 genes annotated in the immune response GO term (GO0006954) if none of the confidence intervals overlapped zero Three genes were significantly downregulated upon the *Emx1*-Cre/*Trim28*-KO and are labeled in red: *Bmp6*, *Cd163*, and *Ptgdr* (P-adj < 0.05), $n = 3$ per condition. Error lines showing 95% confidence intervals. The list of viral defense genes was retrieved from (Liu *et al*, 2018). None of these were significantly different upon the *Emx1*-Cre/*Trim28*-KO.

Source data are available online for this figure.

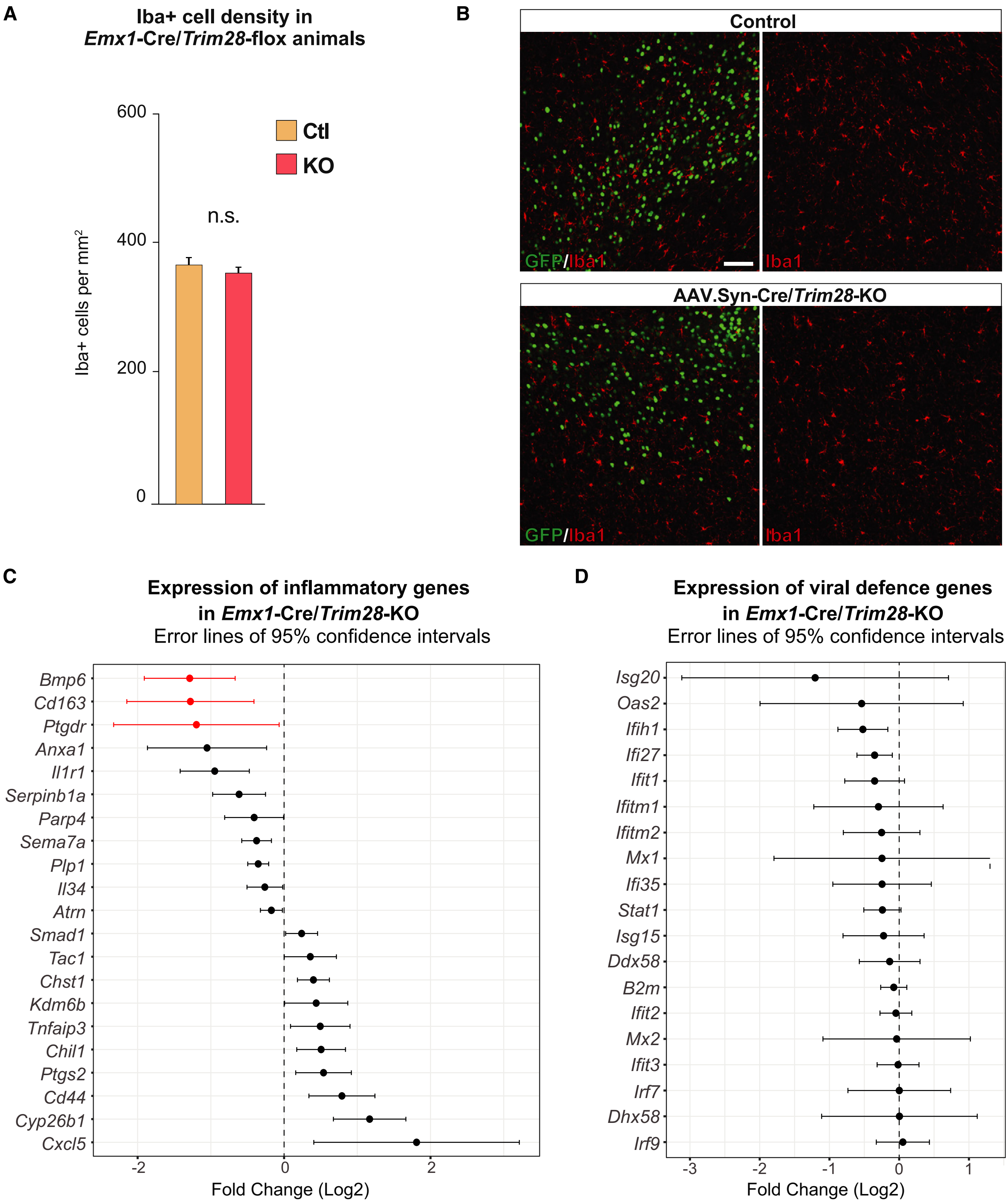

Figure EV5.

 © 2021 The Authors
